# Revealing the Computational Meaning of Neocortical Interarea Signals

**DOI:** 10.3389/fncom.2020.00074

**Published:** 2020-08-18

**Authors:** Hiroshi Yamakawa

**Affiliations:** ^1^University of Tokyo, Tokyo, Japan; ^2^The Whole Brain Architecture Initiative, Edogawa-ku, Japan

**Keywords:** core and matrix thalamocortical circuit, corticocortical circuit, predictive coding, emulation theory of representation, BDI logic, intention, plan

## Abstract

To understand the function of the neocortex, which is a hierarchical distributed network, it is useful giving meaning to the signals transmitted between these areas from the computational viewpoint. The overall anatomical structure or organs related to this network, including the neocortex, thalamus, and basal ganglia, has been roughly revealed, and much physiological knowledge, though often fragmentary, is being accumulated. The computational theories involving the neocortex have also been developed considerably. By introducing the assumption “The signals transmitted by interarea axonal projections of pyramidal cells in the neocortex carry different meanings for each cell type, common to all areas,” derived from its nature as a distributed network in the neocortex, allows us to specify the computational meanings of interarea signals. In this paper, first, the types of signals exchanged between neocortical areas are investigated, taking into account biological constraints, and employing theories such as predictive coding, reinforcement learning, representation emulation theory, and BDI logic as theoretical starting points, two types of feedforward signals (observation and deviation) and three types of feedback signals (prediction, plan, and intention) are identified. Next, based on the anatomical knowledge of the neocortex and thalamus, the pathways connecting the areas are organized and summarized as three corticocortical pathways and two thalamocortical pathways. Using this summation as preparation, this paper proposes a hypothesis that gives meaning to each type of signals transmitted in the different pathways in the neocortex, from the viewpoint of their functions. This hypothesis reckons that the feedforward corticocortical pathway transmits observation signals, the feedback corticocortical pathway transmits prediction signals, and the corticothalamic pathway mediated by core relay cells transmits deviation signals. The thalamocortical pathway, which is mediated by matrix relay cells, would be responsible for transmitting the signals that activate a part of prediction signals as intentions, due to the reason that the nature of the other available feedback pathways are not sufficient for conveying plans and intentions as signals. The corticocortical pathway, which is projected from various IT cells to the first layer, would be responsible for transmitting signals that activate a part of prediction signals as plans.

## 1. Introduction

Understanding the neocortex, which is responsible for cognition and behavior generation in the brain, is of major interest in neuroscience. At the same time, it is useful as a reference for constructing systems with human-like intelligence. The cerebral neocortex, if one considers a large number of areas (dozens, if human) to be nodes, is organized as a hierarchical, distributed network that operates in a highly parallel manner (Felleman and Van Essen, 1991; Mountcastle, 1995; Haberly, 2001; Nassi and Callaway, 2009; Markov et al., 2011, 2014a). The neocortex is also a network of decentralized memory, since each area has its own representation and can maintain its own internal state.

In this interarea network, there are feed-forward paths from lower-order areas that are relatively close to the outer environment to higher-order areas that are more distant, and a feedback path in the opposite direction. Anatomically, most of the former is rostrally directed and most of the latter caudally directed. In addition, in the laminar projection structure, the former mainly originates in the supra- and infra- granular layer and terminates in layer 4 in the higher areas, while the latter mainly originates in the infra granule layer and terminates in the lower area by avoiding layer 4. This opposing directionality brings a hierarchy to the connections among areas (Rockland and Pandya, 1979; Maunsell and van Essen, 1983; Markov et al., 2013, 2014b).

All areas in the neocortex typically have similar microcircuits consisting of six layers, sometime called canonical circuits because of their regularity (Gilbert and Wiesel, 1979; Douglas et al., 1989; Douglas and Martin, 2004; Dhruv, 2015). For computational neuroscience researchers and brain-inspired AI developers, the simplistic hypothesis of a canonical circuit model that treats all areas of the neocortex in the same way is very appealing. The neocortical microcircuit also has the specificity of each area. For example, depending on the change in the thickness of the granular layer in the laminar structure, it is classified as a granular, dysgranular, or an agranular area (Beul and Hilgetag, 2014). It has also been known that the extent to which pyramidal cells receive and integrate inputs, how the inputs are integrated within the dendrites, and the extents of connectivity between neurons vary in different areas (Jacobs and Scheibel, 2002; Elston, 2003; Elston and Fujita, 2014). In light of these facts, there is an argument that the pyramidal cells of the neocortex cannot be a generic component (Luebke, 2017). However, much of the differences among areas are quantitative. Therefore, the possibility of an emergence of a generalized canonical model in the future that treats the differences between areas as differences of parameters has not been ruled out. However, the hurdle to designing such a model in microcircuits, where various types of neurons are densely coupled, is still considerable.

As already mentioned, the neocortex is a distributed network consisting of a number of nodes called areas. In order to achieve communication between nodes, it is necessary to share information representation among them. The following assumptions are plausible for satisfying that requirement.

     Canonical interarea protocol assumption:

The signals transmitted by interarea axonal projections of pyramidal cells in the neocortex carry different meanings for each cell type, common to all areas.

This assumption states that neuron types specify information representations of protocols for interarea communication. The validity of that assumption is discussed below.

In general, a protocol, which is a rule for communication, should be shared among all the nodes to operate a many-to-many communication system. The basic structure of that protocol is a layer composed of a physical transmission channel, an encoding method, identification of a peer, and information representation. In neocortical circuits, there is a common mechanism for the first three: the first transmission pathway is the interarea axon of pyramidal neurons; the second method is realized by neural spiking; the third, identification of a peer, is defined by a connectome.

Therefore, a mechanism to specify information representation is necessary for interarea communication. This communication layer corresponds to the application layer of the TCP/IP model and defines the meanings of the signal being conveyed. In the brain neural circuit, receiving-side neurons can distinguish cell types of sending-side neurons. Therefore, the signals carried by the interarea axonal projections of pyramidal neurons can carry different meanings for each cell type. Moreover, such meanings can be shared across all neocortical areas. There is no other plausible candidate mechanism suitable for sharing information representation through all areas. Therefore, the canonical interarea protocol assumption is plausible. This hypothesis is consistent with the findings of axonal bifurcation (Kennedy and Bullier, 1985; Markov and Kennedy, 2013; Markov et al., 2013), which suggests that groups of FF and FB neurons form a different flow of information.

However, in light of several hypotheses related to the computational function of the neocortex, several types of meanings for signals exchanged between all domains can be identified. In this paper, the meaning of interarea signals is identified as Observation, Deviation, Prediction, Planning, and Intention by comprehensively considering the representative theories of predictive coding (Rao and Ballard, 1999), The emulation theory of representation (Grush, 2004), Reinforcement learning, and BDI logic (Bratman, 1987), taking into account some biological constraints (Detailed discussion in Chapter 2.). Of course, on the neocortex, there are also properties of information that differ from area to area, such as visual, auditory, and motor modalities, but there are orthogonal meanings to the above. For example, if it is visual information, there are visual observation signals, visual deviation signals, visual prediction signals, visual planning signals, and visual intention signals.

In this paper, general meaning of the signals used for interarea communication in the neocortex are identified. Specifically, five theoretically meaningful signals will be assigned to interarea pathways including the corticocortical and thalamocortical circuits, under the canonical interarea protocol assumption.

In the next chapter, several computational theories of neocortical networks for cognition and execution are explained, and the five types of signals required for this network are explained. In chapter 3, the types of connections between areas are summarized, based mainly on anatomical findings of the neocortex and of the thalamus. In chapter 4, anatomical findings and computational findings are integrated by mapping five types of signals to five different pathways. In chapter 5, the proposed hypothesis is discussed from several aspects. Finally, in chapter 6, we summarize the results of this paper.

## 2. Functional Types of Interarea Signals in the Neocortex

In this chapter, we identify five types of signals that are supposed to be exchanged among areas of the neocortex. First, an overview of the five types of signals exchanged among areas of the neocortex will be presented. Taking into account biological constraints, we will show that existing theories, such as predictive coding, reinforcement learning, representation emulation theory, and BDI logic, can support this classification of signals.

### 2.1. A Hypothesis for Five Types of Signals Exchanged Between Areas

**Figure 1:**
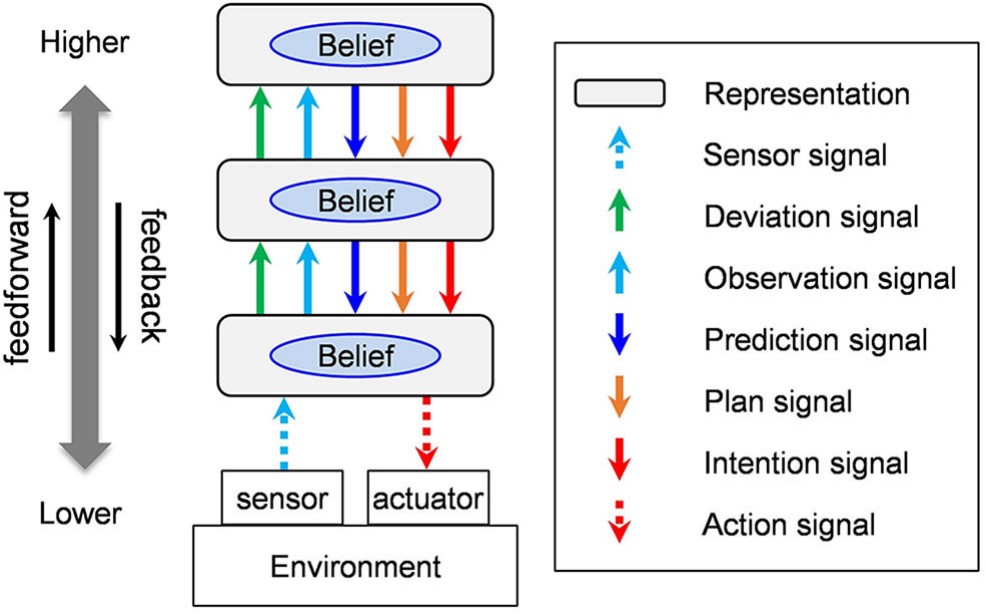
Five types of signals exchanged among areas of the neocortex. In this figure, the complex structure of a real neocortical network is left out in order to show the types of signals that travel in both directions along the hierarchy.

As a preparation, we have provided a brief overview of the basic structure of the neocortex. The neocortex forms a system that consists of hierarchical representations (see Figure 1). Areas that are near sensors that called “lower-order” layers, and those farther from them are called “higher-order” layers. We will call signals that go up the order feedforward signals, and those that go down the order feedback signals. The representation of an area has beliefs, namely, inferred states that correspond to the current sensor and are updated constantly. Here the word “belief” is used in the context of a belief network or in a Bayesian network.

The neocortex is a large, parallel and distributed network system consisting of a large number of hierarchically connected areas. The connections have a complex branching and converging structure that allow for jumping areas while conserving direction (Felleman and Van Essen, 1991; Mountcastle, 1995; Markov et al., 2011, 2014a). In Figure 1, a simple three-level cascade network is drawn as an example, in order to illustrate the classification of signals transmitted between areas in a straightforward manner.

**Observation signals** are feedforward signals that transmit the belief state of a representation toward a higher order. **Prediction signals** are feedback signals that transmit beliefs in a representation to a lower order. **Deviation signals** are feedforward signals that transmit to a higher order the degree of which the belief in a representation deviates from observation signals. **Plan signals** and **intention signals** transmit beliefs that are desirable to be realized. Since the last two types of signals are both prediction signals, they are sent as feedback to a lower order. Plans are part of predictions that are desirable to be realized. Intentions are part of plans that the agent is committing to execute.

### 2.2. Cognition by Discriminative Models

In engineering, cognition systems have traditionally adopted discriminative models. These systems recognize the world by step-by-step processing of inputs, which are observational information obtained by its sensors. In a discriminative model, only observation signals, among the five types of signals mentioned above, are used for information processing. In neuroscience, similarly, it has been generally assumed that observation signals are transmitted feedforwardly through the neocortex hierarchy.

In object cognition of images, the Convolutional Neural Network (CNN) Model, a deep learning method, has been successfully engineered (Yamashita et al., 2018). It is also known that the information representations acquired by CNN models are similar to that of the visual cortex (Yamins and DiCarlo, 2016). In view of the current technical situation, it seems reasonable to assume that in the neocortex, some hierarchical discriminative model that feedforwardly transmits observation signals is implemented.

### 2.3. Cognition by Generative Models

The Bayesian brain hypothesis (Doya et al., 2007; Friston, 2012) regards functions of the neocortex as computations executed on the basis of Bayesian inference. Bayesian inference forms, using an internal model, a probability distribution that can appropriately generate observation signals obtained from sensors.

Predictive coding is a model that uses artificial neural networks and realizes almost the same computational function with Bayesian inference (Rao and Ballard, 1999). This model, too, feedforwardly generates prediction signals so that the signals fit the information obtained by sensors. It then calculates prediction error signals, which are discrepancies between prediction signals and observation signals, and feedforwardly transmits them to modify beliefs.

For the reason discussed below, given the biological constraints, it is difficult to construct a plausible model that recognizes the external world using prediction signals and prediction error signals. We therefore explain how the combination of feedforward signals—observation and deviation signals—and feedback signals—prediction signals—can realize a similar function in a biologically plausible way.

Signal transmission between neurons can only convey positive (or negative) values because of the use of neural activity spikes. However, in order to realize prediction coding, it is necessary to use a prediction error signal with both positive and negative codes. Although the mechanism for transmitting error signals between the neocortical areas has been investigated (Keller and Mrsic-Flogel, 2018), this mechanism is not easy to implement in a biologically plausible way.

Humans, on the other hand, have the ability to show bottom-up attention that keenly detects any unexpected stimulation in the things observed (Treisman and Gelade, 1980; Duncan and Humphreys, 1989; Nothdurft, 1993). It allows us to assume that prediction errors are being dealt with in some way or other in the brain. Thus, we assume that deviation signals, which are not prediction errors themselves but the absolute values of prediction errors, serve as feedforward signals. In the neural circuits of the brain, deviation signals can be implemented naturally since they take only positive values.

If a representation receives a deviation signal that is without a positive nor negative sign, the representation can know that its beliefs should be changed, but it cannot know in what direction its beliefs should be modified. Therefore, using only unsigned deviation signals as feedforward signals in a given representation cannot be an appropriate way for modification of beliefs. Of course, if an observation signal is transmitted as a feedforward signal at the same time, then the representation obtaining it will be able to correct its belief into the right direction. Moreover, as discussed in section 2.2, it is highly plausible to count the observation signal as a kind of feedforward signal. Therefore, it is biologically plausible to use both the deviation and observation signals as feedforward signals.

### 2.4. Execution by Generative Models

In this section, the feedback signals related to generation of execution are construed and summarized by employing background theories such as reinforcement learning, representation emulation theory, and BDI logic.

Reinforcement learning provides an agent some evaluation function in the form of reward, and the agent utilizes it to select actions and maximize the expected reward. Reward is the measure of desirability of things for the agent in question, and though in many cases its setting has to be given externally and in advance, it is possible this setting takes into it other factors, such as the novelty of information obtained and the difference between observation and prediction.

The emulation theory of representation (Grush, 2004), suggests a perspective that generation of action signals is a part of the generation of prediction signals. That is, of all the prediction signals that express some desirable belief state for the agent in the future, the operable ones in some actuators are regarded as actions. This idea has a long history, originating in the Helmholtz machine and feedforward control. The active inference (Friston et al., 2012), which is closely related to predictive coding, can be regarded as a subtype of this theory.

In the light of the above ideas, it can be assumed that the agent selects signals that are expected to be highly rewarding in the future, and outputs the operable part of these signals through the actuator. However, such an agent can only address a single task, and humans, whose daily lives involve multiple tasks, focus on one (or a few) task at a time properly while concentrating resources on it. This form of human behavior is based on the belief-desire-intent (BDI) logic (Bratman, 1987), as well as an implementation as an architecture (Rao et al., 1995). In this theory, the agent retains a series of actions that it believes can realize its goal as a “plan.” Then, the agent selects a plan to commit to and retains it as an “intention.” It also distinguishes between present-directed intentions that can be realized at a certain point in time and future-directed intentions that can be implemented in the future. The affordance competition hypothesis (Cisek, 2007) can be said to be an idea more analogous to BDI logic related to brain mechanisms.

Taken together, reinforcement learning has provided a framework for value-addition using reward in the generation of action. The emulation theory of representation has shown that action is part of prediction, and the BDI logic has distinguished what is committed in the plan as an intention and what is immediately executable in the intention as a present-directed intention. Notably, plans and intentions in BDI theory implicitly include predictions for the future, since they are used by agents when they are feasible.

**Figure 2:**
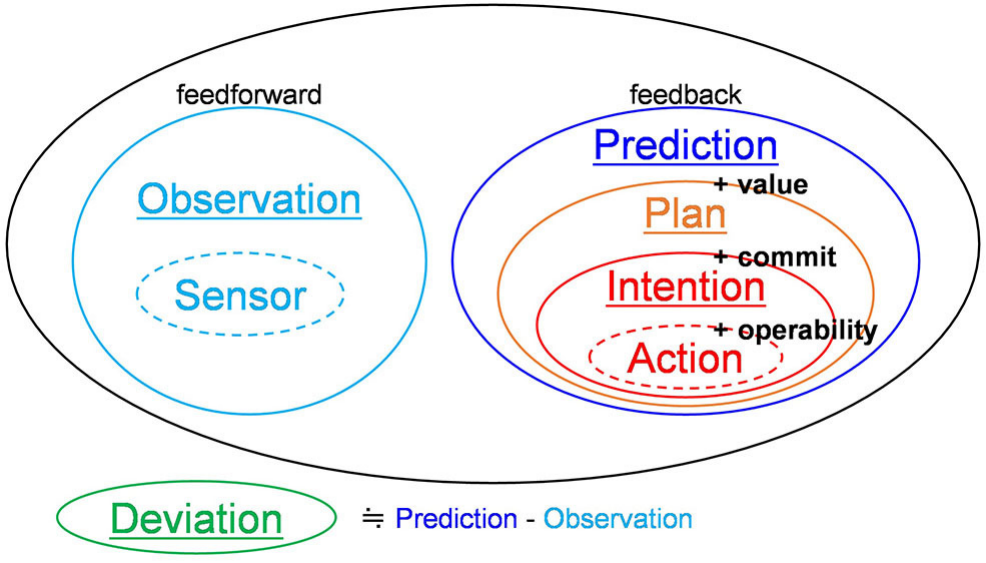
Hierarchy of signals.

Considering the above information, the feedback signals involved in the generation of action can be summarized as follows (see Figure 2). Prediction signals are signals that convey the inferred content of the external world as feedback. Plan signals are a part of prediction signals that convey the content that provides a desirable value to the system. Intention signals are a part of plan signals that convey the content that are committed to execution. Action signals are a part of intention signals that are transmitted to actuators at the present time (Corresponds to the present-directed intentions of BDI logic).

Finally, Figure 2 shows all relevant signals. Feedback signals have been discussed in the previous paragraph. Feedforward signals have been defined in this paper as follows. Observation signals are signals that transmit beliefs about the external world feedforwardly toward a higher-order layer. Sensor signals are a part of observation signals that are directly obtained from sensors. Deviation signals are signals that convey deviation of prediction signals from observation signals. Note that sensor signals and action signals are not exchanged between the neocortex areas. Thus, there are five types of signals exchanged among neocortical areas: observation signals, prediction signals, plan signals, intention signals, and deviation signals.

## 3. Types of Pathways Between Neocortical Areas

**Figure 3:**
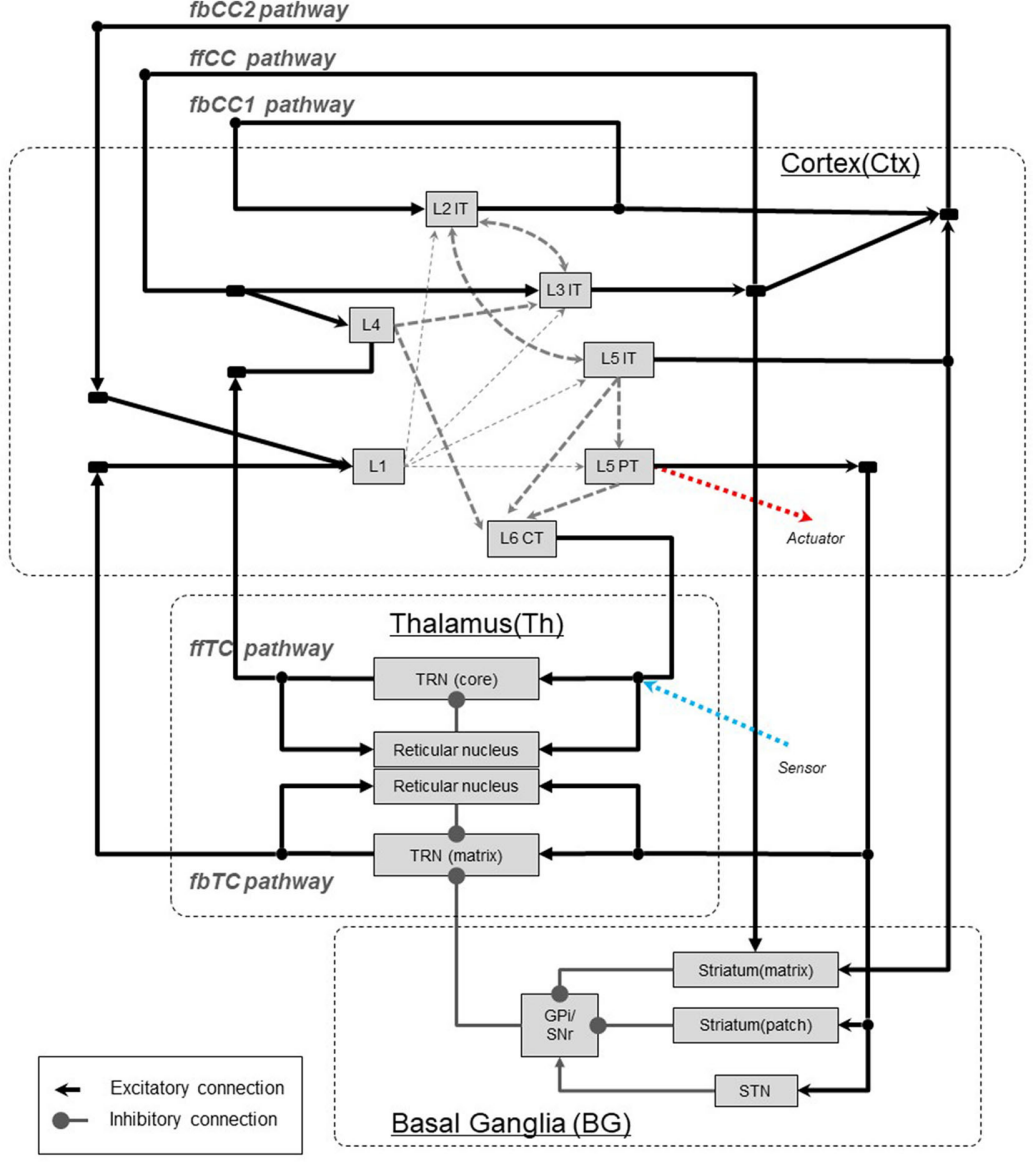
Interarea pathways in the neocortex. The notations ffCC, fbCC, ffTC, and fbTC are used to describe the feedforward/feedback and direct/indirect characteristics of the pathways connecting the neocortical areas. fbCC1, feedback corticocortical pathway 1; fbCC2, Feedback corticocortical pathway 2; ffCC, Feedforward corticocortical pathway; ffTC, Feedforward thalamocortical pathway; fbTC, Top-down thalamocortical pathway. L1, Layer 1; L4, Layer 4; L2IT, Intratelencephalic (IT) neurons in layer 2; L3IT, IT neurons in layer 3; L5IT, IT neurons in layer 5; L5PT, Pyramidal tract (PT) neurons in layer 5; L6CT, Corticothalamic (CT) neurons in layer 6. TRN(core), Core type relay neuron in thalamus; TRN(matrix), Matrix type relay neuron in thalamus; STN, Substantia nigra.

In this chapter, based on anatomical findings for the neocortex and thalamus, all types of interarea pathways will be identified (see Figure 3). Although the internal structures of neocortical local circuits are not of interest in this paper, we review excitatory pyramidal cells, which are the basis of interarea pathways.

Excitatory cells of the neocortex are categorized into three major classes based on differences in axonal projection patterns and information coding (Greig et al., 2013; Shepherd, 2013; Harris and Shepherd, 2015). First, with respect to the corticocortical pathway, we consider it as three pathways that connect areas of the neocortex involving intratelencephalic(IT)-type excitatory cells by direct neural projections. Secondly, for the thalamocortical pathway, we consider it as two pathways, one with corticothalamic (CT)-type neurons of the sixth layer and the other with projections by pyramidal tract (PT)-type neurons of the fifth layer. Thus, we will deal with these five types of pathways that connect areas of the neocortex.

The basal ganglia loop, which is well-known, originates in the neocortex and returns to the neocortex via the basal ganglia and thalamus. However, this pathway performs the function of gating information transfer of the thalamocortical pathway (fbTC pathway), which we will mention later. For this reason, we do not consider it as a pathway that transmits information between areas of the neocortex, and exclude it in this paper.

**Figure 4:**
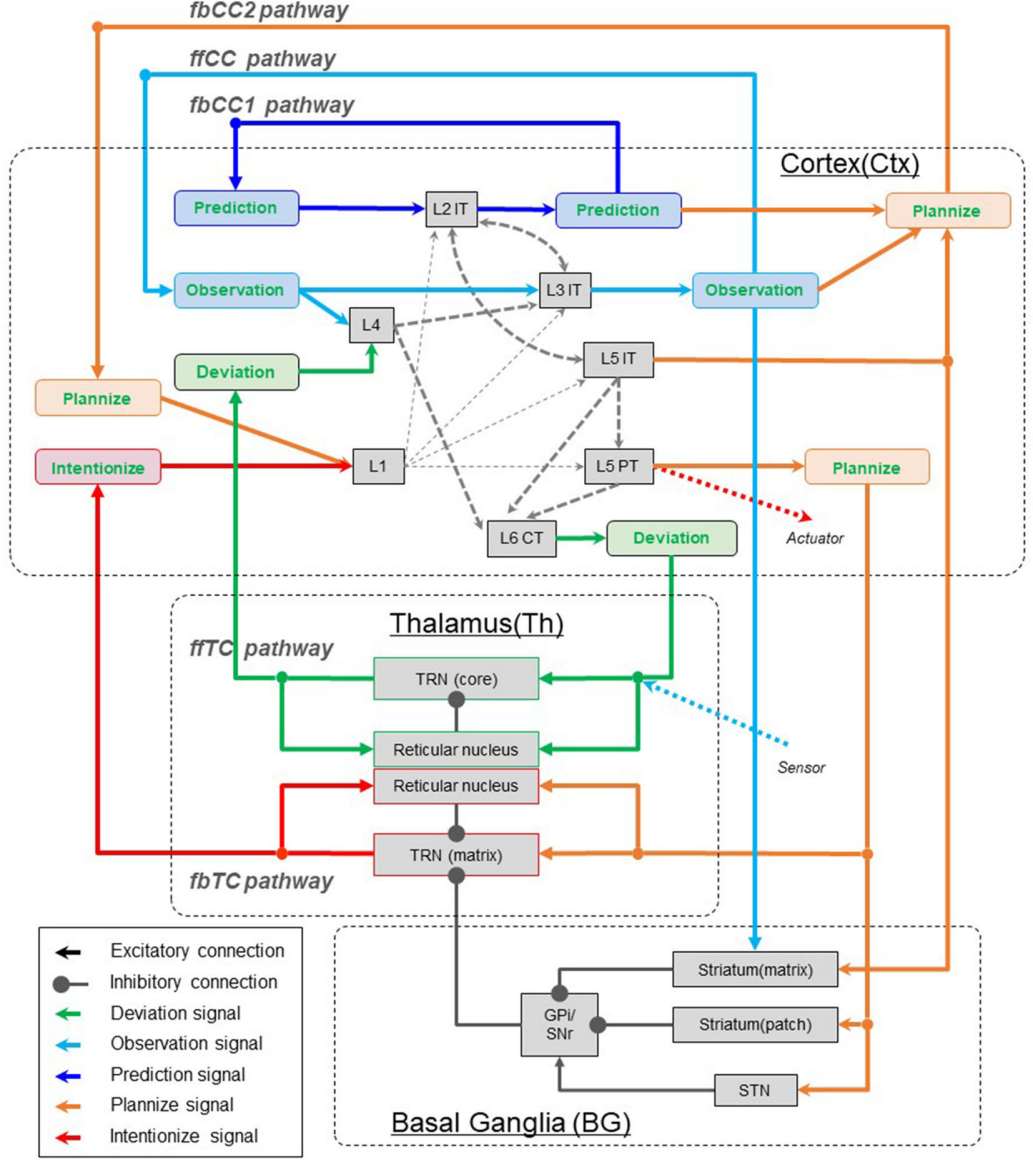
Meaning of signals in interarea pathways. The notations ffCC, fbCC, ffTC, and fbTC are used to describe the feedforward/feedback and direct/indirect characteristics of the pathways connecting neocortical areas. fbCC1, feedback corticocortical pathway 1; fbCC2, Feedback corticocortical pathway 2; ffCC, Feedforward corticocortical pathway; ffTC, Feedforward thalamocortical pathway; fbTC, Top-down thalamocortical pathway. L1, Layer 1; L4, Layer 4; L2IT, Intratelencephalic (IT) neurons in layer 2; L3IT, IT neurons in layer 3; L5IT, IT neurons in layer 5; L5PT, Pyramidal tract (PT) neurons in layer 5; L6CT, Corticothalamic (CT) neurons in layer 6; TRN(core), Core type relay neuron in thalamus; TRN(matrix), Matrix type relay neuron in thalamus; STN, Substantia nigra.

In this paper, the directionality of hierarchy are described as “ff” for feedforward and “fb” for feedback. Furthermore, corticocortical connections are abbreviated to “CC,” and thalamocortical connections are abbreviated to “TC.” For example, the feedforward corticocortical pathway is written “ffCC pathway.” In Figures 3, 4, the characteristic of interarea pathways should be described compactly, since these figures include not only neocortex but also the basal ganglia and the thalamus. Therefore, we use the abbreviations mentioned above to denote the direction of hierarchy and their transit points.

### 3.1. IT Neurons and the Corticocortical Pathway

The largest class of excitatory neurons is intratelencephalic (IT) neurons, which project axons only within the terminal brain (the neocortex, striatum, amygdala, and cortical structures such as claustrum). Projection of IT neurons allows rapid information transfer by forming corticocortical connections that directly exchange signals between different areas, and by utilizing spike timing of neural activity. These IT neurons are the only type of excitatory cells that project to the contralateral cortex (Harris and Shepherd, 2015). Hereinafter, IT neurons in layers 2–6 are abbreviated as L2IT, L3IT, L4IT, L5IT, and L6IT, respectively. In addition, the flow of information among the areas may be controlled by synchronization of activities, and we discuss this possibility later, in the discussion part (ref. 5.3).

L4 IT neurons are special in that they are, in local circuits of the neocortex, located upstream of other IT neurons. Since L4 IT neurons do not have long-range projections toward other areas, they cannot be the starting point of a pathway that connects different areas. Although L6 IT neurons projecting to claustrum form recurrent circuits with each area of the neocortex (Baker et al., 2018), the number of cells in the claustrum is too small to exchange information between areas and is therefore omitted from consideration in this paper.

#### 3.1.1. fbCC1 Pathway

L2IT neurons seem to be functioning as a corticocortical feedback input system (Solari and Stoner, 2011). In macaque monkey experiments, in the V2 and V3 areas, the feedback projections targeting the V1 area is concentrated in the laminar where the L2IT are located (Markov et al., 2014b). This suggests that there is a feedback-type corticocortical connection from higher L2IT to lower L2IT, which we will call the fbCC1 pathway.

#### 3.1.2. ffCC Pathway

L3IT neurons receive projections from L4IT and simultaneously project strongly to L4 IT in other areas of the ipsilateral cortex (DeFelipe et al., 1986; Rockland, 1992; Douglas and Martin, 2004; Barbas et al., 2005; Medalla and Barbas, 2006; Markov et al., 2013). In the granular cortex with the L4 layer, for example, signals projected feedforward from lower areas are transmitted to L3 IT via L4. On the basis of these findings, it is likely that there exists a feedforward corticocortical pathway that conveys the outputs to L4 cells in other areas, which we will call the ffCC pathway. In the early visual cortex, for example, it is a pathway in which information from observations from the external world is transmitted as gamma-band signals toward higher-order areas (Melloni et al., 2007; Bosman et al., 2012; Bastos et al., 2015; Hermes et al., 2015; Michalareas et al., 2016).

L3IT neurons receive not only indirect feedforward projections as described above but also direct feedforward projections from the lower L3IT (Lund et al., 1981; Markov et al., 2014b). It is known from indirect evidence that the activity of the third layer is controlled by the projections from the mediodorsal thalamus (MD) to the anterior cingulate cortex (ACC) in the third layer (Delevich et al., 2015). In the granular cortex, where the L4 layer is largely absent, this direct feedforward pathway would be responsible for most of the signal transmission.

#### 3.1.3. fbCC2 Pathway

Layer 1 of the neocortex, which is also called the molecular layer, has few neurons, and forms connections of axons from outside and dendrites protruding from various pyramidal cells (L2IT, L3, L5IT) in microcircuits. The axons projected into this layer are diverse IT neurons L2IT, L3ITL5IT in a wide range of neocortical areas and matrix-type thalamic relay cells (Felleman and Van Essen, 1991; Pandya and Yeterian, 2003; Markov et al., 2013). It can be supposed that, given these anatomical structures, the projection of specific axons can easily affect or activate different types of pyramidal neurons in microcircuits to control function modules (Solari and Stoner, 2011).

As for the recipient pyramidal cells, because signals received by the dendrites in layer 1 are accumulated as a neural activity potential, the type of neuron from which these signals originated cannot be distinguished. For this reason, it would be reasonable to assume that the signals received in layer 1 fall within a single type of signal as far as their function is concerned. Therefore, in this paper, we call collectively these signals sent to L1 the ffCC2 pathway. These projections would be, neuroanatomically, responsible for activating and modulating information processing in the targeted areas (Solari and Stoner, 2011).

### 3.2. Other Excitatory Neurons and Thalamocortical Pathway

The thalamocortical pathways are an indirect connection of the neocortex. The intervening thalamus can control the flow of information between areas in the neocortex, as pointed out by the Selective Engagement Model (Crosson, 2013) and others. Conversely, however, when this pathway transmits information, there is a delay due to mediation of activities of relay neurons of the thalamus, and also because it is difficult to transmit information using the spike timing of neural activity. In the following subsections, we describe two pathways: the pathway relayed by core relay neurons, and the pathway relayed by matrix relay neurons (Jones, 1998; Piantoni et al., 2016).

#### 3.2.1. ffTC Pathway

The first relay nuclei on the thalamus in the thalamocortical pathway are parvalbumin-positive core thalamic relay cells. Corticothalamic (CT) neurons in the sixth layer that project to these relay cells receive, in the local circuits, projections from a number of IT neurons, including L5 IT, and project mainly to the ipsilateral core-type thalamic relay cells. Core relay neurons project to the fourth layer, targeting a single cortical area (Rodriguez et al., 2004; Bonjean et al., 2012). In addition, reticular nuclei, which receive projections from L6 CT neurons themselves, have inhibitory projections to relay neurons.

Much of the projection from core neurons to the neocortex is consistent with the area receiving the projection and is known to have a recursive structure. At the same time, it is known from studies of visual attention that there is a feedforward indirect pathway through the pulvinar, parallel to the ffCC pathway (Jones, 2001; Shipp, 2003). In this paper, this path is referred to as the ffTC path due to the attention to the feedforward type connection structure.

#### 3.2.2. fbTC Pathway

The second relay nuclei on the thalamus in the thalamocortical pathway are calbindin-positive matrix thalamic relay cells. A subtype of pyramidal tract (PT) neurons (L5 PT) in the fifth layer project to these relay nuclei. This subtype is located downstream of IT neurons in the local circuit, and this group of neurons produces initial preparatory activities that last until the onset of motions (Economo et al., 2018).

Matrix relay cells project mainly to the first layer of a relatively broad neocortical area, simultaneously receiving projections from neocortical L5 PT neurons and inhibitory projections from the basal ganglia (Jones, 1998; Clascá et al., 2012; Mitchell, 2015). Reticular nuclei, which receive projections from L5 PT neurons and relay neurons themselves, have inhibitory projections to relay neurons. GPi/SNR in the basal ganglia dis-inhibit only a few matrix relay neurons at some point in time. Thus, only a few relay cells are allowed to transmit signals at each point in time. This mechanism realizes the gating function of information transmission between areas.

L5 PT cells also project to the striatum (patch). The other sub-type of L5 PT projects to the spinal cord, etc., and outputs pre-action preparatory activities and motor commands.

## 4. Assignment of Signal Meaning for Each Pathway

In this chapter, five computationally meaningful signals (see Chapter 2) are assigned to five pathways (see Chapter 3) that connect the neocortex areas. Through this work, we make computational sense of the signals exchanged through each pathway.

### 4.1. Assignment of Signal Meaning for Feedforward Pathways

For feedforward signals, we observe that it is reasonable to assign observation signals to the ffCC pathway, and deviation signals to the ffTC pathway.

#### 4.1.1. Assign Observation Signal to the ffCC Pathway

Below is the rationale for assigning observation signals to the ffCC pathway.

In order for a system to operate in response to the changing external world, it is desirable to update its beliefs about the state of the world with as little delay as possible. It should also be capable of conveying the information of a rich observation signal. Therefore, a feedforward corticocortical pathway should be assigned to the transmission of the observed signal with less delay and degradation of the information transmission. In other words, it would be reasonable to assign the observed signal to the ffCC pathway.

Neocognitron, which is a visual information processing model, has alternating layers of S cells that extract features of figures and C cells that absorb the misalignment of features. This corresponds to L3 IT cells and L4 cells in the ffCC pathway, as shown in Figure 3. Moreover, given the fact that this model was a prototype in the development of deep learning, it would be reasonable to assign observation signals to the ffCC pathway.

#### 4.1.2. Assign Deviation Signals to the ffTC Pathway

We state the rationale that it is appropriate to assign deviation signals to the ffTC pathway. The simple reason is that the only remaining feedforward path to be assigned is the ffTC pathway.

As mentioned in section 2.3, the bottom-up attention realized by deviation signals involves processes such as selection and gain control of observation signals. These processes would be realized as a modulation on the pathway to receive the observed signal. The terminal end of the ffTC pathway mediated by core relay neurons is suitable for achieving bottom-up attention, for it projects to this fourth layer.

For bottom-up attention to perform its function of selecting the observation signal, the deviation signal must be transmitted a parallel feedforward pathway to the observation signal. It is known that the ffTC path is a feedforward path that is roughly parallel to the ffCC path that conveys the observed information (Murray Sherman and Guillery, 2006). Therefore, it is reasonable to assign a deviation signal to the ffTC pathway.

### 4.2. Assignment of Signal Meaning for Feedback Pathways

As described in chapter 3, there are three types of feedback pathways that connect neocortical areas: fbCC1, fbCC2, fbTC. And, as described in chapter 2, there are three types of signals to be transmitted as feedback: prediction, plan, and intention. However, only the fbCC1 pathway is supposed to be able to transmit these signals without degradation, and the fbCC2 and fbTC pathways are unsuitable. This is because, as already mentioned, the fbCC2 pathway is suitable to play the role of activating and modulating information processing in local circuits. This is also because the fbTC pathway is not suitable for transmitting rich information because it is an indirect pathway that is relayed to the thalamus. Therefore, it is not possible to transmit the prediction, plan, and intention signals among areas using separate paths.

In the process described below, this issue is overcome by the introduction of plannize and intentionize signals. Plannize signals are defined as signals that specify a desirable part of the prediction signal. Intentionize signals are defined as signals that specify a desirable and committed part of the prediction signals. Here, the desired predictions are the belief states that the agent wants to achieve in the future. Since the plan signals are a desirable part of the prediction signals (see section 2.4), the plan signals could be produced by activating a part of the prediction signals with the plannize signals. Since the intention signal is a desirable and committed part of the prediction signals (see section 2.4), intention signals could be produced by activating a part of the prediction signals with intentionize signals.

#### 4.2.1. Assign Prediction Signals to the fbCC1 Pathway

Clearly, from the above discussion, it is appropriate to assign the prediction signals to the fbCC1 pathway.

#### 4.2.2. Assign Intentionize Signals to the fbTC Pathway

We explain below the rationale for assigning the conversion of plan signals into intention signals to the fbTC pathway.

It is traditionally known that in the brain, behavioral decisions at various levels, such as the motor, oculomotor, prefrontal, orbitofrontal, and cingulate levels, are made in the basal ganglia loop that is governed by reward signals (Magrinelli et al., 2016). The basal ganglia loop is often described as consisting of the neocortex-basal ganglia-thalamus-neocortex. However, in actuality, as explained in chapter 3, the GPi/SNR output of the basal ganglia is a circuit that depresses thalamic matrix relay cells on the fbTC pathway.

That is, the fbTC is a pathway that selects, from plan signals generated in various neocortical areas at some point in time, as intention signals that specify currently committed predictions using long-term prediction awards that are calculated at the basal ganglia (Colder, 2011; Yamakawa, 2020).

#### 4.2.3. Assign Plannize Signals to the fbCC2 Pathways

We assign plan signals to the remaining one, the fbCC2 pathway.

The fbCC2 pathway can deliver a variety of signals, including observation and prediction signals. Therefore, it is assumed that the receiving side local circuits learn to control activation or modulation to determine which part of prediction signals should be plannize signals.

Within the neocortical local circuit, the mechanism that determines the part of the prediction signal that is to be carried forward into the plannize signals is not clear. However, it could be assumed that the decision to activate is based on valorization using dopamine projections from the ventral tegmental area. It is assumed that through these learning processes, signals passing through the fbCC pathway will be able to play a plannize role.

### 4.3. Summary of This Section

The overall picture of the mapping of signals with computational meaning derived in Chapter 2 and this chapter to the interarea pathway is shown in Table 1.

**Table 1:** Assignment of meaning to signals in interarea pathways.

**Flow direct**.	**Signals**	**Description of signals**	**Interarea pathway**	**Description of pathway**
Feed-forward	Observation	Signals that feedforward beliefs about the outside world to higher levels of the hierarchy	ffCC	L3IT → L4 or L3IT → L3IT
	Sensor	Signals that are part of the observation signals and are obtained directly from the sensor	–	Sensor → TRN(core)
	Deviation	Signals that convey the deviation of the prediction signals from the observation signals	ffTC	L6CT → TRN(core) → L4
Feed-back	Prediction	Signals that convey an inferred content about the outside world to the feedback	fbCC1	L2IT → L2IT
	Plan	Signals that are parts of prediction signals that are desired to be realized	–	–
	Intention	Signals that are part of the plan signals that are committed to execution	–	–
	Action	Signals that are part of the intention signals and are currently transmitted to the actuator	-	L5PT → Actuator
	Plannize	Signals that specify parts of prediction signals that are desired to be realized	fbCC2	L2IT, L3IT, L5IT → L1
	Intentionize	Signals that specify parts of prediction signals that are desired to be realized and that are committed to execution	fbTC	L5PT → TRN(matrix) → L1

In the supragranular layer, the ffCC pathway transmitting the observed signal and the fbCC1 pathway transmitting the prediction signal are a relatively independent pair (Markov et al., 2014b). This structure is fit for a counterstream theory (Ullman, 1995). In addition, this information flow pair is commonly used in current machine learning: encoding and decoding in deep generative models; inference and generation in the Bayesian networks.

## 5. Discussions

### 5.1. Correspondence of the Thalamic Pathway

The proposed hypothesis is consistent with the following statement by Piantoni et al. (2016) for the thalamus (Piantoni et al., 2016).

“The first role is the one in which sensory information is passed through relatively faithfully to cortical areas, albeit amplified or suppressed based on attentional mechanisms. The second role is to maintain the state of the cortex and to support an appropriate degree of activation, in either wakefulness or sleep.”

The “first role” in the above citation corresponds to the amplification or suppression by the ffTC pathway for the L4, which are terminals of the ffCC pathway that transmit observation signals. This also corresponds to bottom-up attention, which notifies when something in observation signals deviates from prediction signals.

The “second role” involves facilitating neural activities as intentions by transmitting intentionize signals to an area through the fbTC pathway, as intentions that are to be executed in that area. This corresponds to the fact that the feedback attention in the visual cortex transmits attention, which is determined in the frontal lobe according to the tasks to be dealt with, toward the visual cortex as attention signals in the beta band (Buschman and Kastner, 2015).

### 5.2. The Thalamus as the Pathway Responsible for Input to the Telencephalon

In this paper, sensor inputs, which are related to the thalamic pathway, and actuator outputs hold a special position, since we focused on information exchange in areas in the neocortex. However, for information processing in the telencephalon, the role of the thalamus as an input pathway is rather essential. Typically, thalamic core neurons are most strongly associated with sensory relays, such as the lateral geniculate nucleus (Jones, 1998; Clascá et al., 2012).

In Figure 4, sensor inputs from the external world appear like they are causing an interruption into the TRN(core) in the ffTC pathway. However, in terms of phylogenetic development, this pathway may convey sensor inputs to L4 neurons in the primary sensory cortex has preceded, and the function of L6 CT neurons to transmit deviation signals are a posterior addition to that.

The pyramidal tract, which transmits action signals from L5 PT neurons in the motor cortex to the spinal cord, is indispensable as an output stream for the telencephalon. The fbTC pathway would be a part of this pathway that has been developed as the pathway that projects to L1 via TRN (matrix). This pathway is utilized in the basal ganglia of the cerebrum, which appear only in vertebrates in terms of phylogenetic development. Probably even before the basal ganglia emerged, plannize signals had been transmitted through the fbCC2 pathway that transmits from L5 IT directly to L1 layers in other areas. However, this pathway does not allow selection based on delayed reward and timing-control of plans to be executed. The basal ganglia and the fbTC pathway were probably added to improve this point.

Therefore, both the role of the fbTC pathway in transmitting deviation signals, and the role of the fbTC pathway in transforming plan signals into intention signals, are likely to have been added later in terms of phylogeny.

### 5.3. Dynamic Connection by Synchronous Coupling of the ffTC/fbTC Pathways

Although the corticocortical pathway is a direct connection that has sufficient capacity for transmitting observation and prediction signals, this pathway has difficulty, as the other side of it, in dynamically controlling the flow of information. To address this weakness, a mechanism called the synchronous coupling mechanism has been proposed (Canolty et al., 2006; Lakatos et al., 2008).

Relying on the following reasons, the anatomical structure of the thalamus is suitable for driving synchronization of neural activities in a wide area of the neocortex. First, relay cells project axons toward all areas of the neocortex. Second, the structure of the thin nerve sheet, which surrounds the thalamus and is made of inhibitory Reticular nuclei, has a structure that is suitable to induce synchronized activity of the whole (or rather a wide part) of the thalamus. The feedback of the reticular nucleus to the thalamus is related to the overall synchronization of thalamic neurons in slow-wave phases of sleep and in a certain form of epilepsy (Steriade et al., 1993; Timofeev et al., 2012).

Models in which the thalamus produces brain waves that appear in the spectrum from sleep to wakefulness, such as delta, alpha, beta, and gamma waves, have also been explored (Li et al., 2017), and the main brain waves in wakefulness are low gamma waves of about 40 Hz (=25 ms) and beta waves. Vibrations in the beta band increase feedback signals, and vibrations in the gamma band increase feedforward signals (Buschman and Kastner, 2015). In hearing, electrical stimulation to the reticular nucleus of rats evokes gamma-band oscillations (40 Hz) in the auditory cortex (Macdonald et al., 1998). In a visual space selection task, an increase of synchronization in alpha waves in the interconnected visual cortical areas (V4 and TEO) occurs from the connection between a part of the thalamus (pulvinar) and cortical areas (Saalmann et al., 2012).

Based on these findings, there is a possibility that the coupling mechanism, which is mentioned above, is realized by the coordination of both pathways, one involving low-frequency oscillations (alpha or slow beta waves), which act as top-down attention, and the other involving high-frequency oscillations (gamma waves) of the ffTC pathway, which act as bottom-up attention, in the reticular nucleus of the thalamus.

## 6. Conclusions

The neocortex is a system that recognizes and generates actions by a hierarchical network of representation. In this paper, two presuppositions are assumed. First, a neocortex consists of areas formed by uniform local circuits. Second, different pathways that connect areas transmit different meaning signals.

Several theories, such as predictive coding, reinforcement learning, representation emulation theory, and BDI logic, are used as starting points for the study, and signals exchanged between domains are classified into five types, taking into account biological constraints. The following types of signals have been defined: observation signals that transmit information about the external world feedforwardly, prediction signals that transmit inference about the external world as feedback, deviation signals that transmit deviation of observation signals from prediction signals feedforwardly, plan signals that transmit desirable prediction to the system as feedback, and intention signals that transmit prediction which are both desirable and committed to its realization as feedback.

Then, based on the anatomical knowledge of the neocortex and the thalamus, we identified the pathways connecting areas as three types of corticocortical pathways and two types of thalamocortical pathways. Then, as to the pathway for feedforward, we reckoned that the corticocortical pathway, which originates at IT cells in the third layer (L3IT) in an area and projects to cells in the higher-order fourth layer(L4), transmits observation signals, and the thalamocortical pathway, which originates at cells in the corticothalamic cells in the sixth layer(L6CT) in an area and connects to cells in the higher-order fourth layer via thalamic core relay cells, transmits observation signals. Of the three feedback pathways that exist, the one with the properties required to convey prediction signals is the corticocortical pathway, which originates at IT cells of the second layer(L2IT) of an area and connects to IT cells in the lower-order second layer. Therefore, we assigned prediction signals to this pathway.

Thus, the thalamocortical pathway projecting from fifth-layer pyramidal tract cells (L5PTs) in the area through thalamic matrix relay cells to the lower first layer is assigned an intentional signal that activates a part of the predicted signal as an intention. The plannize signal, which activates a part of the prediction signals as plans, was assigned to cortical pathways that project from various IT cells in areas to the first layer in lower-order areas.

In this paper, based on the above examination, a hypothesis that provides computational meaning to the five types of interarea signals in the neocortex was proposed. In the future, further examinations would be required to reveal the meaning of signals exchanged in brain organs such as the neocortex, thalamus, and basal ganglia from the perspectives of anatomy, physiology, and computational theory, based on their meanings in this hypothesis.

In this paper, study of projections from the layer 6 pyramidal neurons, are limited only for projection to the matrix-type relay cells in the thalamus (ffTC pathway). There exists various subtypes of pyramidal neurons in layer 6 (Thomson, 2010; Narayanan et al., 2017; Radnikow and Feldmeyer, 2018). Pyramidal neurons are known to project to the first and sixth layers of the lower area of the neocortex, as well as into the striatum and claustrum (Rockland and Van Hoesen, 1994; Markov et al., 2014b; Baker et al., 2018). In the future, investigations for specifying computational meanings (probably the Deviation-like) and projection targets for every subtype of pyramidal neuron in layer 6 should be addressed.

## Data Availability Statement

The raw data supporting the conclusions of this article will be made available by the authors, without undue reservation.

## Author Contributions

The author confirms being the sole contributor of this work and has approved it for publication.

## Conflict of Interest

The author declares that the research was conducted in the absence of any commercial or financial relationships that could be construed as a potential conflict of interest.
